# Dissemination and genetic analysis of the stealthy *vanB* gene clusters of *Enterococcus faecium* clinical isolates in Japan

**DOI:** 10.1186/s12866-018-1342-1

**Published:** 2018-12-13

**Authors:** Yusuke Hashimoto, Jun Kurushima, Takahiro Nomura, Koichi Tanimoto, Kiyoko Tamai, Hideji Yanagisawa, Komei Shirabe, Yasuyoshi Ike, Haruyoshi Tomita

**Affiliations:** 1 Department of Bacteriology, Gunma University Graduate School of Medicine, 3-39-22 Showa-machi, Maebashi, Gunma 371-8511 Japan; 2 Laboratory of Bacterial Drug Resistance, Gunma University Graduate School of Medicine, 3-39-22 Showa-machi, Maebashi, Gunma 371-8511 Japan; 3 MIROKU Medical Laboratory Inc, 659-2 Innai, Saku, Nagano, 384-2201 Japan; 4 Yamaguchi Prefectural Institute of Public Health and Environment, 2-5-67 Aoi, Yamaguchi, Yamaguchi 753-0821 Japan

**Keywords:** VRE, Antibiotic resistance, Outbreak, Conjugation, Reversion

## Abstract

**Background:**

VanB-type vancomycin (VAN) resistance gene clusters confer VAN resistances on *Enterococcus* spp. over a wide range of MIC levels (MIC = 4–1000 mg/L). However, the epidemiology and the molecular characteristics of the VAN susceptible VanB-type *Enterococcus* still remain unclear.

**Results:**

We characterized 19 isolates of VanB-type *Enterococcus faecium* that might colonize in the gut and were not phenotypically resistant to VAN (MIC = 3 mg/L). They were obtained from two hospitals in Japan between 2009 and 2010. These isolates had the identical *vanB* gene cluster and showed same multilocus sequence typing (MLST) (ST78) and the highly related profiles in pulsed-field gel electrophoresis (PFGE). The *vanB* gene cluster was located on a plasmid, and was transferable to *E. faecium* and *E. faecalis*. Notably, from these VanB-type VREs, VAN resistant (MIC≥16 mg/L) mutants could appear at a frequency of 10^− 6^–10^− 7^/parent cell in vitro. Most of these revertants acquired mutations in the *vanS*_*B*_ gene, while the remainder of the revertants might have other mutations outside of the *vanB* gene cluster. All of the revertants we tested showed increases in the VAN-dependent expression of the *vanB* gene cluster, suggesting that the mutations affected the transcriptional activity and increased the VAN resistance. Targeted mutagenesis revealed that three unique nucleotide substitutions in the *vanB* gene cluster of these strains attenuated VAN resistance.

**Conclusions:**

In summary, this study indicated that stealthy VanB-type *E. faecium* strains that have the potential ability to become resistance to VAN could exist in clinical settings.

**Electronic supplementary material:**

The online version of this article (10.1186/s12866-018-1342-1) contains supplementary material, which is available to authorized users.

## Background

**Fig. 1:**
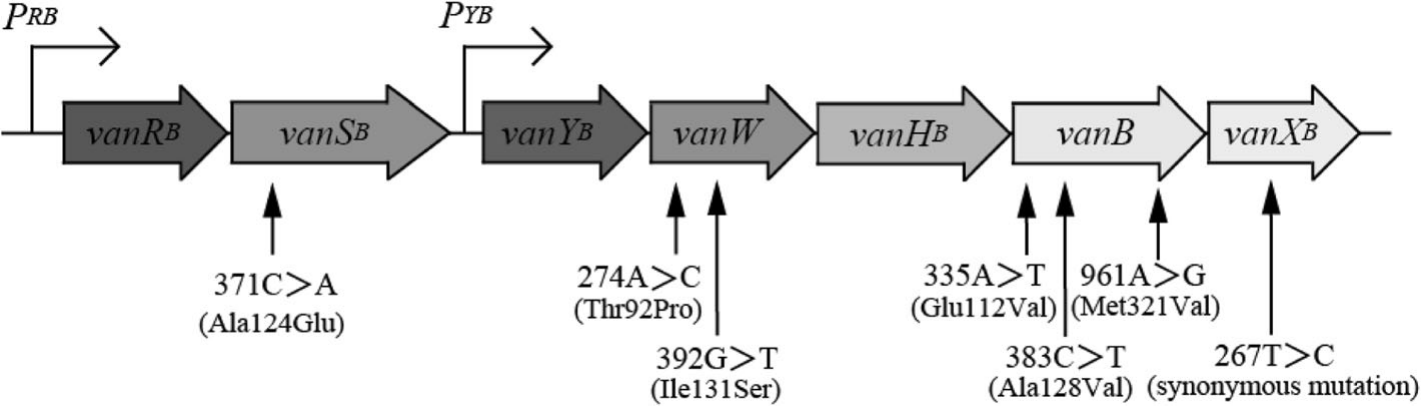
Schematic representation of the *vanB* gene cluster and single nucleotide variations in M1 and Y7 compared with the *vanB* gene cluster in BM4661. Expression of the *vanB* cluster genes is regulated by the two-component regulatory system of VanS_B_/VanR_B_. In the presence of VAN, the histidine kinase VanS_B_ senses VAN and activates the transcriptional activator VanR_B_. Consequently, the two-component regulatory genes (*vanR*_*B*_ and *vanS*_*B*_) and the resistance genes (*vanY*_*B*_, *vanW*, *vanH*_*B*_, *vanB* and *vanX*_*B*_) are transcribed from the VanR_B_-driven promoters *PR*_*B*_ and *PY*_*B*_, respectively, to mediate the VAN resistance [8]. The arrows indicate the location of nucleotide variations (and resulting amino acid substitutions) identified in the *vanB* gene cluster of M1/Y7 compared with that of BM4661

*Enterococcus* spp. is a typical opportunistic pathogen causing urinary tract infections, bloodstream infections and surgical site infections in compromised hosts. Vancomycin (VAN) is a glycopeptide antibiotic that inhibits peptidoglycan synthesis and is used to treat severe Gram-positive bacterial infections [1]. VAN resistance clusters were distributed among several enterococcal species [2]. Notably, the most of VAN resistant enterococci (VRE) are *Enterococcus faecali*s and *Enterococcus faecium*. VRE was first reported in England and France in 1986 [3, 4] and is now one of the major nosocomial pathogens in the world [5]. Due to the limited options for treatment, invasive infections by this pathogen are important causes of mortality. For that reason, the active surveillance is being carried out to prevent the spread of VRE [6]. The VAN resistance in VRE are classified into eight acquired gene clusters. These are *vanA*, *vanB*, *vanD*, *vanE*, *vanG*, *vanL*, *vanM* and *vanN* [2]. VanA- and VanB-type VRE make up of a majority of VRE and most important in clinical settings. VanA-type VRE shows a high resistance to both VAN (MIC = 64–100 mg/L) and teicoplanin (TEC) (MIC = 16–512 mg/L), whereas VanB-type VRE shows a susceptibility to TEC (MIC = 0.5–1 mg/L) and various levels of resistance to VAN (MIC = 4–1000 mg/L) [6]. The *vanB* gene cluster consists of a two-component regulatory system (*vanR*_*B*_, *vanS*_*B*_) and five resistance genes (*vanY*_*B*_, *vanW*, *vanH*_*B*_, *vanB, vanX*_*B*_) (Fig. 1) [7]. Contrary to the highly conserved resistance genes, the amino acid sequences of VanS_B_ and VanR_B_ show less similarities to those of VanS and VanR (*vanA* gene cluster) with 34 and 23% identities, respectively [8]. These differences are suspected to be responsible for the characteristics of VanB-type resistance [8]. Outbreaks of VanB-type VRE occurred in Europe, USA and worldwide [5, 9, 10]. The wide range of VAN resistance of VanB-type VRE (MIC = 4–1000 mg/L) makes it more difficult to detect in clinical settings. A previous study reported that 55% of VanB-type VRE isolated in an outbreak in the neonatal ICU of a German hospital showed low MICs of VAN (less than 4 mg/L) [9]. Meanwhile, there is concern that such low-level VRE may cause treatment failure due to its conversion to a high level of resistance [11]. Indeed, it has been reported that low-level VAN resistant *E. faecium* became VAN resistant during antibiotic therapy and this was named vancomycin-variable *E. faecium* (VVE) [12–14]. In Japan, the first VRE was VanA-type *E. faecium* isolated in 1996 [15] and the first outbreak of VRE was caused by VanB-type *E. faecium* [16]. Although the prevalence of VRE is presumed to be low in Japan in comparison with those in other countries, little is known about the prevalence of VanB-type VRE with low-level VAN resistance. In this study, we characterized *E. faecium* isolates that harbored the *vanB* gene clusters but were not resistant to VAN (MIC = 3 mg/L), and performed genetic analysis to assess the responsible determinants for the attenuated VAN resistance of their *vanB* gene clusters.

## Results

### Isolation of VAN susceptible *E. faecium* harboring the *vanB* gene cluster

**Fig. 2:**
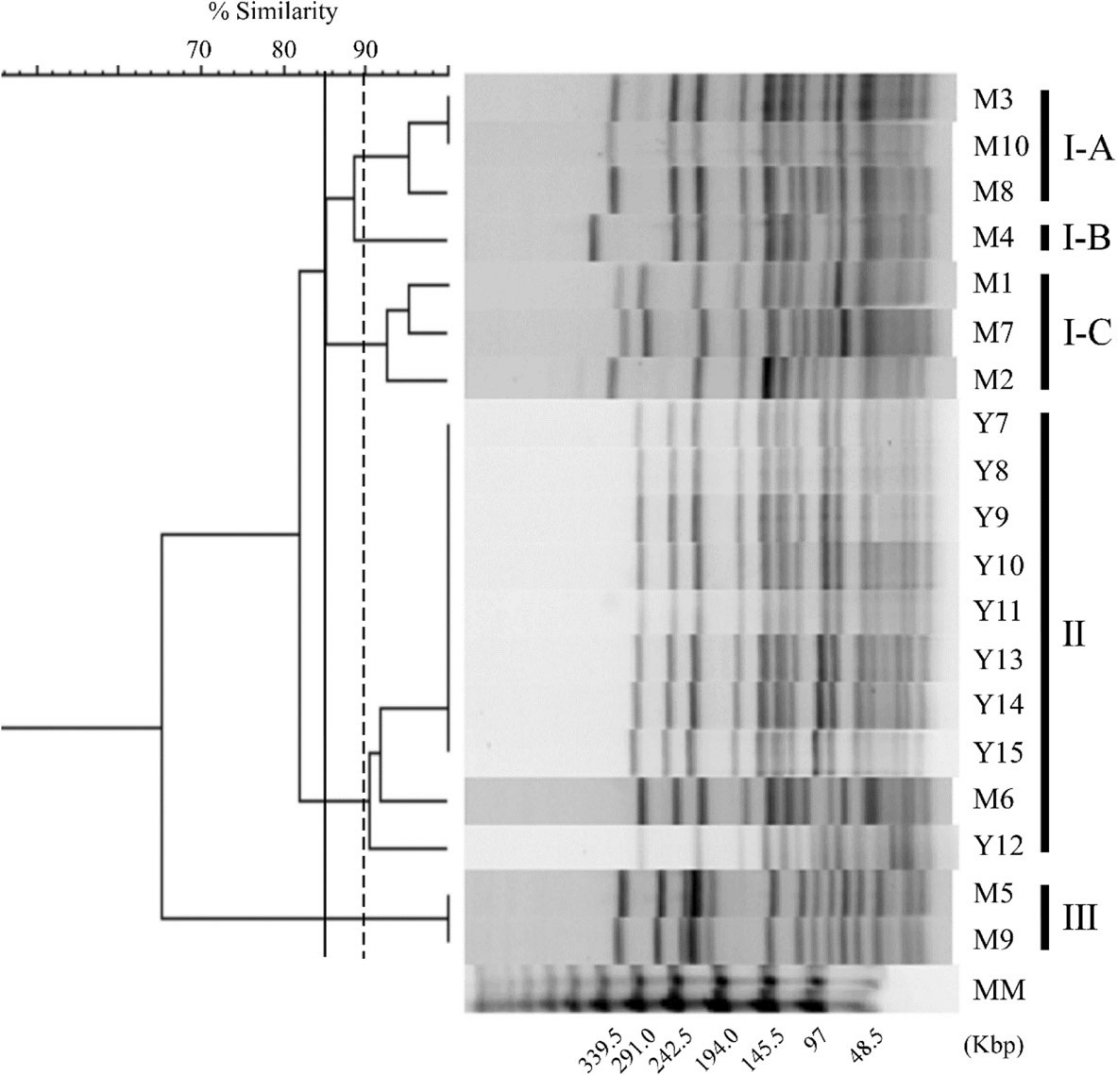
PFGE profiles and dendrogram of the VAN susceptible VanB-type *E. faecium* isolates. Bacterial DNAs were digested with *Sma*I and separated by pulsed-field gel electrophoresis (PFGE). The genetic relatedness was analyzed using the Dice coefficient and the dendrogram was constructed with the clustering algorithm of Unweighted Pair-Group Method with an Arithmetic Mean (UPGMA) using FP Quest Software (Bio-Rad). The optimization and the tolerance were 1 and 1.5%, respectively. Major clusters and subclusters of the isolates were delineated with 85 and 90% similarity cutoff values for PFGE as indicated by the vertical solid line and dotted line, respectively. A lambda PFG Ladder (New England BioLabs, MA) was used as the Molecular Marker (MM)

In 2009 and 2010, a total of nineteen VAN susceptible *E. faecium* strains that harbored the *vanB* gene cluster were isolated from the feces of individual in-patients during a VRE screening test at two hospitals located in adjacent prefectures in Japan. We designated ten isolates as M1–M10 from Hospital A and nine as Y7–Y15 from Hospital B, respectively (Table 1). All the patients did not show any significant symptoms of bacterial infectious diseases and were carriers. The VanB-type VAN resistance genes were detected by PCR. All of the 19 isolates were susceptible to VAN (MIC = 3 mg/L) and TEC (MIC = 1 mg/L), and resistant to ampicillin (MIC≥128 mg/L), tetracycline (MIC = 64 mg/L), erythromycin (MIC> 256 mg/L) and ciprofloxacin (MIC = 64 mg/L) (Table 1). To determine the correlation between their genetic backgrounds, we performed PFGE and MLST analysis (Fig. 2, Table 1). Based on the results from PFGE analysis, these 19 isolates were classified into three main clusters (I, II, and III) with an 85% similarity value as cutoff point. Clusters I was further divided into three sub-clusters (I-A, I-B, and I-C) based on a 90% of similarity value. Y7–Y15 strains obtained from hospital B were all categorized into cluster II, showing that they were highly related in PFGE pattern. Compared with the Y-series strains, M-series strains obtained from hospital A showed wider genetic variation, although they were clustered and had more than 85% similarity values. MLST showed that these 19 isolates belonged to a ST78 lineage (allelic profile, 15–1–1-1-1-1-1), indicating that all these VAN susceptible *E. faecium* strains with the *vanB* gene cluster had a similar genetic background, but there was a slight genetic variation.

**Table 1:** Bacterial strains used in this study

Strain/plasmid	Species	Source	Date of Isolation (mo/yr)	Hospital	VRE genotype	Multilocus sequence typing	PFGE types	MIC (mg/L)^*a*^									
								VAN	TEC	AMP	GEN	KAN	STR	CHL	TET	ERY	CIP
M1	*E. faecium*	feces	09/09	A	VanB	ST78	I-C	3	1	256	> 1024	> 1024	32	8	64	>256	64
M2	*E. faecium*	feces	10/09	A	VanB	ST78	I-C	3	1	256	> 1024	> 1024	32	8	64	>256	64
M3	*E. faecium*	feces	10/09	A	VanB	ST78	I-A	3	1	256	> 1024	> 1024	32	8	64	>256	32
M4	*E. faecium*	feces	10/09	A	VanB	ST78	I-B	3	1	256	8	> 1024	32	8	64	>256	64
M5	*E. faecium*	feces	10/09	A	VanB	ST78	III	3	1	128	8	128	256	8	64	>256	64
M6	*E. faecium*	feces	10/09	A	VanB	ST78	II	3	1	256	8	> 1024	32	8	64	>256	64
M7	*E. faecium*	feces	10/09	A	VanB	ST78	I-C	3	1	256	> 1024	> 1024	32	8	64	>256	64
M8	*E. faecium*	feces	10/09	A	VanB	ST78	I-A	3	1	256	> 1024	> 1024	32	8	64	>256	64
M9	*E. faecium*	feces	10/09	A	VanB	ST78	III	3	1	128	8	128	256	8	64	>256	64
M10	*E. faecium*	feces	0/09	A	VanB	ST78	I-A	3	1	256	> 1024	> 102	32	8	64	>256	64
Y7	*E. faecium*	feces	02/10	B	VanB	ST78	II	3	1	256	> 1024	> 1024	32	8	64	>256	64
Y8	*E. faecium*	feces	02/10	B	VanB	ST78	II	3	1	256	> 1024	> 1024	32	8	64	>256	64
Y9	*E. faecium*	feces	02/10	B	VanB	ST78	II	3	1	256	> 1024	> 1024	32	8	64	>256	64
Y10	*E. faecium*	feces	02/10	B	VanB	ST78	II	3	1	256	> 1024	> 1024	32	8	64	>256	64
Y11	*E. faecium*	feces	02/10	B	VanB	ST78	II	3	1	256	> 1024	> 1024	32	8	64	>256	64
Y12	*E. faecium*	feces	02/10	B	VanB	ST78	II	3	1	128	> 1024	> 1024	32	8	64	>256	64
Y13	*E. faecium*	feces	03/10	B	VanB	ST78	II	3	1	256	> 1024	> 1024	32	8	64	>256	64
Y14	*E. faecium*	feces	03/10	B	VanB	ST78	II	3	1	256	> 1024	> 1024	32	8	64	>256	64
Y15	*E. faecium*	feces	03/10	B	VanB	ST78	II	3	1	256	> 1024	> 1024	32	8	64	>256	64
FA2–2	*E. faecalis*	*–*	*–*	*–*	*–*	*–*	*–*	1	1	2	32	128	64	8	2	0.5	1
FA2–2/pMG2200	*E. faecalis*	*–*	*–*	*–*	VanB	*–*	*–*	64	1	2	32	128	64	8	2	0.5	1
M1TC^*b*^	*E. faecalis*	*–*	*–*	*–*	VanB	*–*	*–*	3	1	2	> 1024	> 1024	64	8	64	0.5	1
Y1TC^*b*^	*E. faecalis*	*–*	–	*–*	VanB	–	–	3	1	2	> 1024	> 1024	64	8	64	0.5	1
BM4105RF	*E. faecium*	*–*	*–*	*–*	*–*	*–*	*–*	1	1	3	8	> 1024	32	2	1	0.5	2
M1TC2^*c*^	*E. faecium*	*–*	*–*	*–*	VanB	–	–	3	1	3	> 1024	> 1024	32	2	128	0.5	2
Y7TC2^*c*^	*E. faecium*	*–*	*–*	*–*	VanB	–	–	3	1	3	> 1024	> 1024	32	2	128	0.5	2

^a^VAN, vancomycin; TEC, teicoplanin; AMP, ampicillin; GEN, gentamicin; KAN, kanamycin; STR, streptomycin; CHL, chloramphenicol; TET, tetracycline; ERY, erythromycin; CIP, ciprofloxacin. ^*b*^Corresponding transconjugants of FA2–2 obtained by filter mating with the donor M1 or Y7 strain, respectively. ^*c*^Corresponding transconjugants of BM4105RF obtained by filter mating with the donor M1 or Y7 strain, respectively

### Single nucleotide variations (SNVs) in *vanB* gene cluster of VAN susceptible isolates

To identify the determinants for attenuated VAN resistance in the M1–M10 and Y7–Y15 strains, we analyzed the nucleotide sequences of the *vanB* gene clusters in M1 and Y7, which were the first isolates detected in the respective hospitals (Fig. 1, Table 2). The nucleotide sequences of the *vanB* gene clusters of these strains were completely identical. Then we compared the nucleotide sequences of the *vanB* gene clusters of M1 and Y7 with that of VanB-type *E. faecium* BM4661, which shows a high-level resistance to VAN (MIC = 256 mg/L) [17]. Compared with the *vanB* gene cluster of BM4661, M1 and Y7 had the same nucleotide variations leading to six amino acid substitutions in the *vanB* gene cluster (Fig. 1, Table 2). Similarly, we compared the sequences of these isolates with those of other VanB-type VRE in the NCBI database displaying MIC-values of VAN greater than 16 mg/L [16–21]. Despite various levels of resistance to VAN, those strains showed high sequence identities in the *vanB* gene cluster (Additional file 1: Table S1). However, a total of three unique substitutions in VanS_B_ (Ala124Glu), VanW (Thr92Pro) and VanB (Ala128Val) were detected in M1 and Y7 (Fig. 1, Table 2, Additional files 2, 3 and 4: Figures S1–3).

**Table 2:** Single nucleotide variations found in the *vanB.* gene cluster of *E. faecium* M1 and Y7

Gene	Substitutions^*b*^	
	BM4661^*a*^ > M1/Y7	
*vanS*_*B*_	371C > A	(Ala124Glu)^*d*^
*vanW*	274A > C	(Thr92Pro)^*d*^
*vanW*	392G > T	(Ile131Ser)
*vanB*	335A > T	(Glu112Val)
*vanB*	383C > T	(Ala128Val)^*d*^
*vanB*	961A > G	(Met321Val)
*vanX*_*B*_	267 T > C	SM^*c*^

^*a*^The genetic information for BM4661 (accession no.; FJ767776.1) was obtained from the genome database in NCBI (http://www.ncbi.nlm.nih.gov/). ^*b*^The nucleotide sequences of *vanB* gene clusters of M1 and Y7 strains were identical. The brackets indicate amino acid substitutions in *vanB* gene clusters. The numbers of substitutions represent the location of each gene and protein. ^*c*^SM, synonymous mutation. ^*d*^Unique substitutions for M1 and Y7 strains (Additional file 2-4: Figures S1–3)

### The *vanB* gene cluster of M1 or Y7 is mobilized to *E. faecalis and E. faecium*

To test the effect of the host genetic background on the VAN resistance, conjugative transfer experiments of the *vanB* gene cluster were carried out. The *vanB* gene cluster of M1 or Y7 was not transferred by broth mating (data not shown), but was successfully transferred by filter mating to the recipient strain *E. faecalis* FA2–2 together with gentamicin, kanamycin and tetracycline resistances, and to *E. faecium* BM4105RF together with gentamicin and tetracycline resistances (Table 1). The resistance gene against kanamycin was expected to be co-transferred to BM4105RF. However, we could not detect this because BM4105RF is intrinsically resistant to kanamycin. The *vanB* gene clusters of M1 and Y7 transferred at frequencies of around 10^− 6^ to 10^− 7^ per donor cell to FA2–2, and around 10^− 7^ to 10^− 8^ per donor cell to BM4105RF by filter mating. We identified the nucleotide sequences of the *vanB* gene clusters of the representative transconjugants (recipient; FA2–2), designated as M1TC (donor; M1) or Y7TC (donor; Y7). It was confirmed that the sequence of the *vanB* gene clusters in M1TC or Y7TC and that of the respective donor strain were identical. The transconjugants showed low MIC values for VAN and TEC as well as the donor strain (Table 1), indicating that neither the *vanB* gene cluster from M1 nor Y7 confers the glycopeptide resistance to the FA2–2 and the BM4105RF strains or to the original *E. faecium* host.

### The *vanB* gene cluster of M1 or Y7 is located on a plasmid and transferred to a chromosome of the recipient

To determine the localization of the *vanB* gene cluster in these strains, we performed Southern transfer and a hybridization analysis. Southern hybridization of the *vanB* gene probe to the ca. 330 kb and ca. 100 kb S1 nuclease-treated DNAs of M1 and Y7 indicated that *vanB* gene cluster located on a plasmid (Additional file 5: Figure S4). However, co-hybridization with the *vanB* gene probe and 23S rRNA gene probe to I-*Ceu*I-digested DNAs were identified in M1TC and Y7TC (Additional file 6: Figure S5). Furthermore, we could not confirm the transfer of the original plasmid to M1TC or Y7TC by Southern hybridization analysis (Additional file 5: Figure S4). These results strongly suggested that the mobile elements including the *vanB* gene cluster were inserted into the chromosomes of the transconjugants.

### Reversion of the low-level VAN resistance of *vanB* gene cluster to the higher-level VAN resistance

**Fig. 3:**
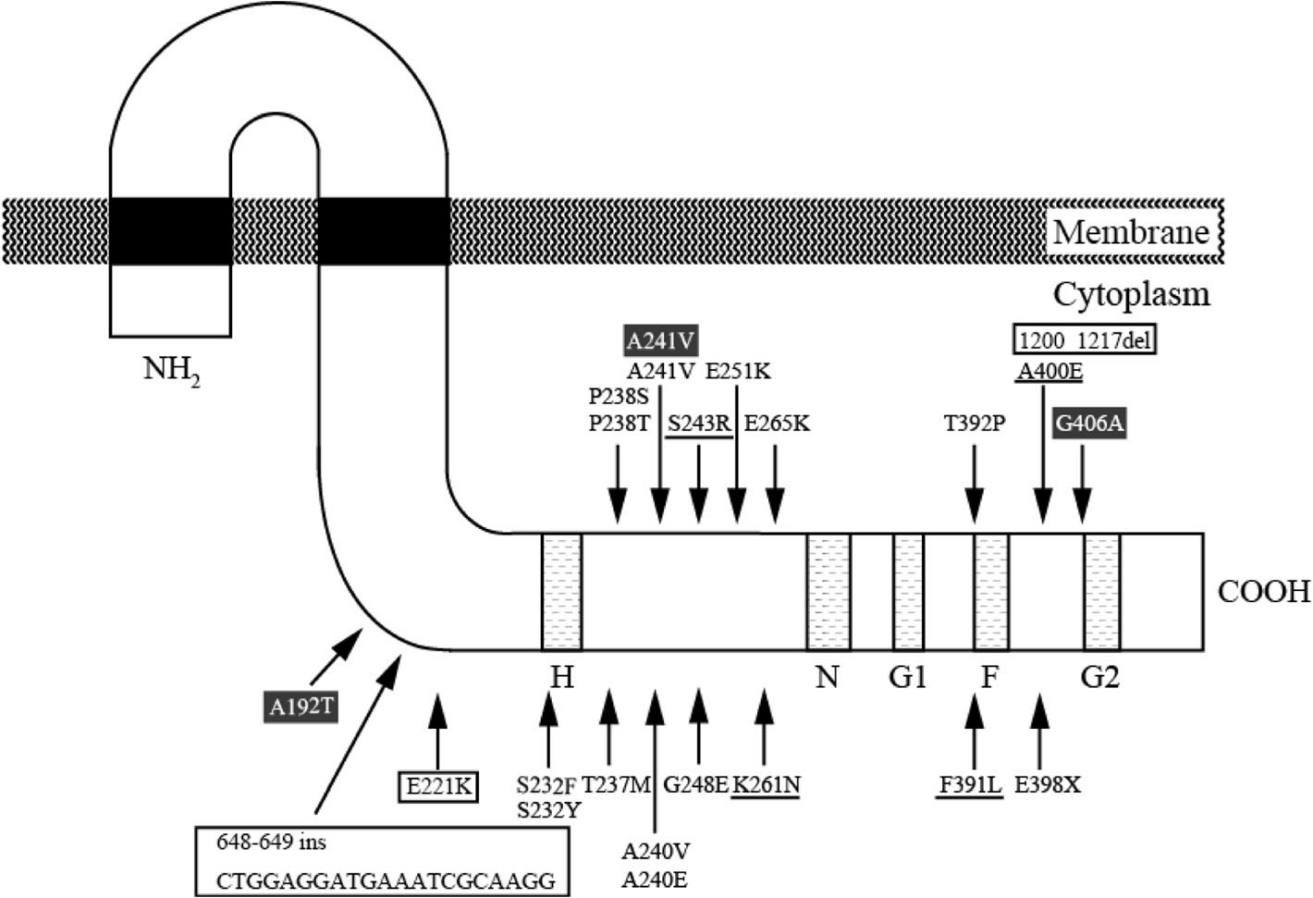
Schematic representation of novel mutations in the *vanS*_*B*_ gene of the revertants. VanS_B_ contains the motifs designated as H, N, G1, F, G2 (lightly shaded boxes) that are the conserved motifs of histidine protein kinases [44]. The His residue (233) in the H box is the primary autophosphorylation site [29].The arrows and characters in black boxes represent mutations found in M1R, the underlined characters represent those in Y7R, those in boxes represent mutations in M1TCR and characters by themselves represent mutations in Y7TCR, respectively

To test whether the *vanB* gene cluster in M1 and Y7 was able to revert VAN susceptible phenotype to VAN resistant phenotype, we performed reversion experiments. Zero point one milliliter of each 24 h culture of M1, Y7, M1TC and Y7TC strains without drug was plated onto a THB agar plate containing VAN at concentration of 16 mg/L, incubated at 37 °C. Spontaneous VAN-resistant mutants were obtained with a frequency of 10^− 6^–10^− 7^ /cell (Table 3). We defined one spontaneous VAN-resistant derivative mutant as a revertant. The frequencies of reversion in the transconjugants M1TC and Y7TC were almost as same as those of M1 and Y7. To examine the genetic changes in the *vanB* gene cluster, we first checked the nucleotide sequence of the *vanB* gene cluster of the 14 revertants derived from M1TC or Y7TC, transconjugants from mating with FA2–2 as a recipient. Ten revertants derived from M1TC and four from Y7TC were designated as M1TCR1–M1TCR10 and Y7TCR1–Y7TCR4, respectively. The mutations found in the *vanB* gene cluster in these revertants were concentrated in the intracytoplasmic domain of VanS_B_ (Fig. 3, Table 4). Four out of ten revertants from M1TC (40%) and three out of four from Y7TC (75%) had mutations in the *vanS*_*B*_ gene. All revertants showed higher MIC values for VAN (MIC> 16 mg/L). Among them, M1TCR6 was the only strain showing both the VAN and TEC resistant phenotype. This revertant had an insertion of 21 bp (7 a. a.) in the intracytoplasmic domain of VanS_B_ (Fig. 3, Table 4). Furthermore, in two revertants, M1TCR2 and Y7TCR4, mutations were identified in *vanB* gene. We also examined the sequences of the intracytoplasmic domain of the *vanS*_*B*_ gene in the 24 revertants derived from M1 and Y7 (M1R1─M1R17 and Y7R1─Y7R7). Of these revertants, 17 revertants (71%) had mutations in the intracytoplasmic domain of VanS_B_ (Fig. 3, Additional file 7: Table S2). We could not obtain revertants with mutations in the *vanR*_*B*_, *vanW*_*B*_, *vanH*_*B*_ or the *vanX*_*B*_ genes. The *vanS*_*B*_ gene encodes the sensor protein of the two-component regulatory system regulating transcription levels of the *vanB* gene clusters. This result suggested that the VAN resistant phenotype was caused by a change in the transcription level of the *vanB* gene cluster.

**Table 3:** Frequency of reversion to VAN-resistant phenotype

Parent strain	The frequency of reversion^*a*^
M1	(4.2 ± 2.0) × 10^–7^
Y7	(1.7 ± 1.2) × 10^−6^
M1TC^*b*^	(2.4 ± 0.5) × 10^−7^
Y7TC^*b*^	(3.5 ± 1.0) × 10^–7^

^*a*^Frequency was estimated from the colony-forming unit (CFU) ratio of resistant strains to total strains. ^*b*^Corresponding transconjugants of M1 or Y7 strain

**Table 4:** Glycopeptide MICs and detected mutations in *vanB* gene cluster of the VAN-resistant revertants

Strain	Mutation^*a*^		MIC (mg/L)^*b*^		RT-PCR (Fold change (±SE)^*c*^	
	*vanS*_*B*_ gene	*vanB* gene	VAN	TEC	VAN (−)	VAN (+)
M1TC	–	–	3	1	N. D.	1
M1TCR1	N. D.	N. D.	16	1	N. D.	4.4 (±1.0)
M1TCR2	N. D.	574G > A(Ala192Thr)	16	1	N. D.	3.6 (±0.5)
M1TCR3	661G > A(Glu221Lys)	N. D.	16	1	N. D.	5.8 (±1.2)
M1TCR4	N. D.	N. D.	16	1	–	–
M1TCR5	N. D.	N. D.	16	1	N. D.	4.9 (±1.4)
M1TCR6	648-649 ins CTGGAGGATGAAA TCGCAAGG	N. D.	128	128	36.1 (±8.0)	39.5 (±13.9)
M1TCR7	1200–1217 del	N. D.	16	1	–	–
M1TCR8	N. D.	N. D.	16	1	–	–
M1TCR9	N. D.	N. D.	16	1	–	–
M1TCR10	661G > A(Glu221Lys)	N. D.	32	1	–	–
Y7TC	–	–	3	1		
Y7TCR1	722C > T(Ala241Val)	N. D.	16	1	–	–
Y7TCR2	N. D.	N. D.	16	1	–	–
Y7TCR3	574G > A(Ala192Thr)	N. D.	64	1	–	–
Y7TCR4	1218G > C(Gly406Ala)	317C > A(Ala106Glu)	16	1	–	–

^*a*^The sequence was compared with the vanB gene cluster of the cognate parent strain, M1TC or Y7TC. ^*b*^VAN: vancomycin, TEC: teicoplanin. ^*c*^Real-time PCR data represent the fold changes in *vanX*_*B*_ transcriptional level relative to that of M1TC in the presence of vancomycin (1 mg/L; VAN (+)). The values were the means of three independent experiments with standard error, each experiment performed in duplicate. N. D.; not detected

### Increased expression level of the *vanB* gene cluster in the revertants

The transcription of the *vanB* gene cluster is strictly suppressed in the absence of VAN, and induction of transcription by VAN via the VanS_B_/R_B_ pathway is essential for mediating VAN resistance [7]. To test whether the increased VAN resistance was due to the increase of transcription in *vanB* gene cluster, we examined the transcription level of *vanX*_*B*_ gene as a representative of the *vanB* gene cluster by real-time PCR (Table 4). In the absence of VAN, transcription of *vanX*_*B*_ was not detected in all strains except M1TCR6. In the presence of VAN, there was an approximately 4-fold increase in M1TCR1, M1TCR2 and M1TCR5 compared with that in a parent strain M1TC. There was ca. 6-fold increase in M1TCR3 and ca. 40-fold increase in M1TCR6 compared with that in M1TC. Additionally, the transcription of *vanX*_*B*_ gene in M1TCR6 was detected even in the absence of VAN (Table 4). These data suggested that the increased expression levels of the *vanB* gene cluster were associated with the increased VAN resistance of the revertants, although no mutation was identified in *vanB* gene cluster in M1TCR1 and M1TCR5.

### Acquisition of the *vanB* gene cluster did not have effect on the growths of transconjugants and revertants

**Fig. 4:**
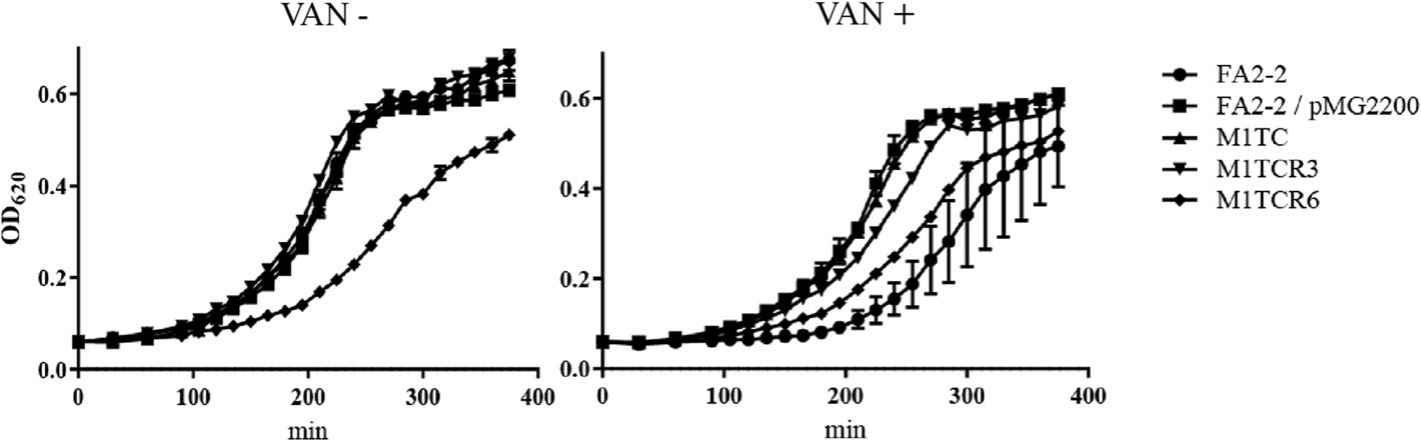
Growth curves of the recipient, transconjugant and revertants in the presence or absence of VAN. Overnight bacterial cultures were diluted 100-fold in fresh THB broth with (Right) or without (Left) 1 mg/L VAN. The culture was incubated at 37 °C and the turbidity was measured at an optical density of 620 nm (OD_620_) at each time point. The values were the mean of three independent experiments with standard error, each experiment was performed in triplicate

The drug resistant determinants impose a burden on cell growth, and the biological cost for expressing the drug resistance is an important factor for their prevalence in host human tissue or any clinical setting [22, 23]. To examine the effect of the *vanB* gene clusters with various level of VAN resistances on bacterial growth, FA2–2, FA2–2/pMG2200, M1TC, M1TCR3 and M1TCR6 were individually incubated at 37 °C in THB and their growth curves were analyzed (Fig. 4). Individual growth curves were similar to each other, except for M1TCR6 in the absence of VAN. Similarly, growth speed of M1TCR6 was lower than those of other VanB-type enterococci in the presence of VAN. These results indicated that the acquisition of the mobile element including the *vanB* gene cluster did not suppress bacterial growth in vitro, if its gene expression was inducible (and not constitutive).

### Targeted mutagensis of *vanB* gene cluster restored the resistance to VAN

To examine whether single nucleotide variations (Fig. 1, Table 2) in the *vanB* gene clusters are responsible for the attenuated VAN resistance (in M1 and M7), we carried out targeted mutagenesis by homologous recombination with the mutated genes using a recombinant plasmid pCJK47.

Three unique substitutions, in VanS_B_ (371C > A; Ala124Glu), VanW (274A > C; Thr92Pro) and VanB (383C > T; Ala128Val), were detected in M1 and Y7 (Table 2). We exchanged these three unique nucleotide variations to those of VanB-type VRE displaying MIC values of VAN greater than 16 mg/L (Table 2, Additional file 2-4: Figures S1–3). The mutant strain with VanW (274C > A; Pro92Thr) and/or VanB (383 T > C; Val128Ala) did not show increased VAN resistance (MIC = 3 mg/L). However, the MIC values of VAN in the mutants with VanS_B_ (371A > C; Glu124Ala) and VanW (274C > A; Pro92Thr) or VanB (383 T > C; Val128Ala) were increased (MIC = 8 mg/L). Exchanges of all these three nucleotides in M1TC restored the MIC value of VAN up to 16 mg/L (Table 5). In particular, VanS_B_ (371A > C; Glu124Ala) appeared to be the key substitution required for restoration of VAN resistance (Table 5).

**Table 5:** Glycopeptide MICs of the MITC derivatives that had been introduced mutations in *vanB* gene cluster

Strain	MIC (mg/L)^*a*^	
	VAN	TEC
M1TC	3	1
M1TC *vanS*_*B*_ _E124A^*b*^	4	1
M1TC *vanB_* V128A^*b*^	3	1
M1TC *vanW_* P92T^*b*^	3	1
M1TC *vanS*_*B*_*_* E124A/*vanB_* V128A^*b*^	8	1
M1TC *vanS*_*B*_*_* E124A/*vanW_* P92T^*b*^	8	1
M1TC *vanB_* V128A/*vanW_* P92T^*b*^	3	1
M1TC *vanS*_*B_*_ E124A/*vanB_* V128A/*vanW_* P92T^*b*^	16	1

^*a*^VAN, vancomycin; TEC, teicoplanin ^*b*^Three unique substitutions for M1 and Y7

255 strains were exchanged to that of the reference gene of pMG2200 by pCJK47-used 256 homologous recombination

## Discussion

### Epidemiology of M1–M10 and Y7–Y12 *E. faecium* strains

In this study, we genetically characterized the *E. faecium* isolates M1–M10 and Y7–Y15 strains, which were susceptible to VAN despite possessing the *vanB* gene cluster (Table 1). These strains were isolated from two unrelated hospitals, but showed similar genetic backgrounds belonging to ST78 (Fig. 2, Table 1). The ST78 is one of the important groups which is associated with hospitalized patients and belongs to Bayesian analysis of population structure (BAPS) 2–1. The BAPS 2–1 is a hospital-adapted lineage [24]. The PFGE patterns of the Y7–Y15 strains were highly related, suggesting the clonal dissemination of these isolates in Hospital B. Meanwhile, M1–M10 strains showed a relatively small genetic variation in their PFGE pattern, and the PFGE of M5 and M9 (cluster III) showed 65% similarities with those of the other M-series strains. The recombination occurs more frequently and has an important role in a genetic diversity in *E. faecium* [24, 25]. During a long term surveillance, it was reported that de novo generation of vancomycin-resistant *E. faecium* by mobile element including *vanB* gene cluster was frequent [26]. Taken together with the fact that M1–M10 and Y7–Y15 strains shared an completely identical *vanB* gene cluster and similar antimicrobial susceptibility profile, there seemed to be a possibility of a horizontal gene transfer and/or a long-term colonization sufficient for genetic rearrangement in Hospital A and a transmission between patients in Hospital B.

### Stealth behavior of potential VAN resistant enterococci

About a hundred cases of VRE infection are reported in Japan annually by National Institute of Infectious Disease (NIID) surveillance but there are few investigations of the prevalence of VRE (http://www.nih.go.jp/niid/ja/). Matsushima et al. reported the regional spread of VRE in Kyoto prefecture [27]. However, because of the VAN-concentration used for the VRE screening (15 mg/L), it is likely that they failed to detect VAN susceptible VanB-type enterococci [27]. Under the Infectious Disease Control Law in Japan (Kansenshō-Hō, Act on Prevention of Infectious Disease and Medical Care for Patients Suffering Infectious Disease of Japan), notifiable VRE is defined as enterococcal clinical isolates causing infectious disease and showing VAN MIC≥16 mg/L. On the other hand, MIC clinical breakpoints for Enterococci defined by Clinical and Laboratory Standards Institutes (CLSI) are as follows, for vancomycin (mg/L), susceptible, ≤4; intermediate, 8–16; resistant, ≥32 (http://clsi.org/). The *E. faecium* isolates we reported here showed VAN susceptible phenotype (MIC = 3 mg/L) and they are not notifiable VRE in Japan (Table 1). In addition, we also demonstrated the spontaneous reversion of the VAN susceptible *vanB*-carrying *E. faecium* from VAN susceptible phenotype to VAN resistant phenotype. These revertants, displaying MIC values of VAN greater than 16 mg/L, were considered intermediate or resistant by CLSI definition. European Committee on Antimicrobial Susceptibility Testing for enterococci (EUCAST) defined vancomycin resistant, > 4 mg/L. In either case, vancomycin treatment might not be effective against these revertants. This spontaneous reversion occurred with a frequency of 10^− 6^–10^− 7^/parent cell (Table 3). This frequency seems to be relatively high in comparison with that of spontaneous antibiotic-resistant mutants in other bacteria [28]. These barely detectable VAN susceptible strains have the capacity for long-term colonization in the human gut without producing any symptoms, because this *vanB* gene cluster does not affect bacterial fitness in the absence of VAN (Fig. 4). Consistent with our results, Marie-Laure Foucault et al. has reported that inactivated or inducible Tn*1549* carrying the *vanB* gene cluster produces no additional fitness cost [23]. Furthermore, the transfer of this *vanB* gene cluster from the plasmid residing in M1 or Y7 to the chromosome of other enterococcal cell indicated the risk of efficient dissemination of VAN resistance genes. Therefore, it should be considered that “stealth behavior” of this *vanB* gene cluster should be subjected to the active surveillance by the standard VAN screening method in clinical settings.

### Genetic mutations leading to the reversion phenotype

It has been reported elsewhere that enterococcus strains harboring the VanB-type VAN resistance gene cluster showed various MIC level for VAN or TEC [6, 7]. However, the determinants involved in this variation of MIC are not well understood [9]. We showed the experimental reversion to increased VAN resistance by targeted mutagenesis in M1 and Y7 strains, and in their corresponding transconjugants M1TC and Y7TC (Tables 1, 3). The reversion to the increased VAN resistance appeared to be resulted from the increased expression level of the *vanB* gene cluster (Table 4). It was previously reported that various mutations in *the vanS*_*B*_ gene changed the glycopeptide resistance phenotype [4, 7, 29]. Consistent with this, 24 strains of the 38 revertants (63%) that were tested carried the novel mutations in intracytoplasmic domain of *vanS*_*B*_ gene (Fig. 3, Table 4 and Additional file 7: Table S2). In general, the high frequency of reverting to antibiotics resistance and the concentration of mutations in “one gene” indicated that the mechanism of reversion was loss of function of the gene rather than gain of function [28]. These mutations appeared to lead to the attenuated phosphatase function of VanS_B_. Meanwhile, the rest of the revertants derived from M1TC or Y7TC did not have any mutation in the *vanB* gene cluster and the sensor domain of *vanS*_*B,*_ except for M1TCR2 and Y7TCR4 carrying the mutations in the *vanB* gene (Table 4). However, two revertants, M1TCR1 and M1TCR5 did not have mutation in *vanB* gene cluster, but still showed increased expression of the *vanB* gene cluster in the presence of VAN (Table 4), indicating that there must be mutation(s) outside of the *vanB* gene cluster affecting the transcription of *vanB* genes. Additionally, M1TCR2 had an amino acid substitution in the *vanB* gene (Table 4). According to previous reports, this amino acid substitution of VanB did not seem to be important for D-Ala-D-Lac ligase function [30–32]. Therefore, the responsible mutation for reversion of M1TCR2 could be outside of the cluster like that of M1TCR1 and M1TCR5. The possible cross talks between VanR_B_ and cognate enterococcal kinase were presumed to be responsible in the previous study [33]. This report may provide clues about the mechanism of reversion without a mutation in the *vanB* gene cluster including the *P*_*RB*_/*P*_*YB*_ promotor region. A further study is underway to clarify this point. Collectively, reversion from an attenuated VAN resistance mediated by this *vanB* gene cluster to an increased VAN resistance appears to be associated with the increased transcription of the *vanB* gene cluster, but the mutation or the mechanism responsible for the reversion appears to be varied and not limited in the *vanB*-related genes.

### Single nucleotide variations in the *vanB* gene cluster attenuated resistance to VAN

In addition to this revertant analysis, we carried out targeted mutagenesis to examine whether the single nucleotide substitutions (Table 2) in the *vanB* gene cluster of M1 and Y7 are responsible for the attenuated resistance to VAN. There were three unique amino acid substitutions (Ala124Glu in VanS_B_, Thr92Pro in VanW and Ala128Val in VanB) in the M1/Y7 strain (Table 2). It was reported that the essential genes of VanB-type VAN resistance were *vanH*_*B*_, *vanB* and *vanX*_*B*_ [1] and deletion of *vanW*_*B*_ in V583 did not affect the VAN resistance [34]. In addition, it has been reported previously that the amino acid substitution in VanB (Ala128Val) did not have an impact on ligase function [30–32]. However, our experiment suggested that three single nucleotide variations resulting in an amino acid change in the *vanB* gene cluster appeared to have a combined impact on vancomycin resistance (Table 5). The MIC-values of VAN in the mutant strains without VanS_B_ (371A > C; Glu124Ala) were equal to that in the parent strain (MIC = 3 mg/L). Therefore, the nucleotide sequence variation in *vanS*_*B*_ gene is assumed to be the key factor, but all of the three variations are needed for VAN susceptible phenotype.

## Conclusions

It has been thought that VVE means only VanA-type VAN susceptible enterococci that can revert to the resistant phenotype [14]. As shown in this study, these VAN susceptible VanB-type isolates also have the ability to revert to the highly resistant phenotype to VAN. Due to the potential threat in clinical settings and the risk of treatment failure, these strains should be included in VVE. Further study is required to understand by what molecular mechanism(s) the stealthy *vanB* gene cluster converts to the higher-level resistance and the responsible determinant(s) for the varied range of glycopeptide MIC in the VanB-type resistance enterococci.

## Methods

### Bacterial strains, plasmids, growth condition, oligonucleotides, media, and antimicrobial reagents

The bacterial strains and plasmids used in this study are shown in Table 1. *E. faecium* M1–M10 strains and Y7–Y15 strains were provided by MIROKU Medical Laboratory Co. (Nagano, Japan) and Yamaguchi Prefectural Institute of Public Health and Environment, respectively. pMG2200 is the pheromone-responsive plasmid isolated from *E. faecalis* clinical isolates harboring the *vanB* gene cluster. pMG2200 conferred VAN resistance on the host strain, showing an MIC for VAN of more than 64 mg/L [16]. The oligonucleotides used in this study are shown in Additional file 8: Table. S3. Enterococcal strains were routinely grown in Todd-Hewitt broth (THB; Difco, Detroit, MI) at 37 °C. *Escherichia coli* strains were grown in Luria-Bertani (LB; Difco) at 37 °C. All antibiotics were obtained from Sigma Co. (St. Louis, MO).

### Antibiotic susceptibility test

MICs were determined by the agar dilution method according to Clinical and Laboratory Standards Institutes (CLSI) guidelines (http://clsi.org/). After each strain was grown overnight in Mueller-Hinton broth (MHB; Nissui, Tokyo, Japan), the cultures were diluted 100-fold with fresh MHB. An inoculum of approximately 5 × 10^5^ cells (5 μl) was spotted onto a series of Mueller-Hinton agar (Eiken, Tokyo, Japan) plates containing a range of concentrations of the test drug. After incubation at 37 °C for 24 h, the susceptibility was determined. The interpretation of the results was in compliance with standards recommended by CLSI. The breakpoints of MICs for resistance to antibiotics were defined as follows (mg/L); vancomycin (VAN), ≥16; teicoplanin (TEC), ≥16; ampicillin (AMP), ≥12.5; gentamicin (GEN), > 500; kanamycin (KAN), ≥1024; streptomycin (STR), > 1000; chloramphenicol (CHL), ≥32; tetracycline (TET), ≥16; erythromycin (ERY), ≥8; ciprofloxacin (CIP), ≥4 (Table 1). *E. faecalis* V583 and ATCC29212 were used as controls.

### Pulsed-field gel electrophoresis (PFGE) analysis and dendrogram

PFGE analysis was performed as previously described [35]. Briefly, enterococci DNA embedded in an agarose plug was digested overnight at 25 °C using *Sma*I (Roche, Basel, Switzerland), and then subjected to PFGE using a CHEF-MAPPER (Bio-Rad, CA) according to the manufacture’s protocol. The guidelines proposed by Tenover et al. were used for the interpretation of PFGE results [36]. The genetic relatedness was analyzed using the Dice coefficient and the dendrogram, and was calculated with the clustering algorithm of Unweighted Pair-Group Method with an Arithmetic Mean (UPGMA) using FP Quest Software (Bio-Rad) [37, 38]. A lambda PFG Ladder (New England BioLabs, MA) was used as the Molecular Marker (MM).

### Southern transfer and hybridization analysis

PFGE analyses with S1 nuclease or I-*Ceu*I were performed as described above. Briefly, enterococci DNA embedded in agarose plug was digested for 20 min at 37 °C with S1 nuclease (Promega, WI) or overnight at 37 °C with I-*Ceu*I (New England BioLabs, MA), the DNAs were then subjected to PFGE using a CHEF-MAPPER (Bio-Rad, CA) according to the manufacture’s protocol. Southern hybridization was performed with the digoxigenin-based non-radioisotope system of Boehringer GmbH (Mannheim, Germany), and Southern transfer and the hybridization procedure were carried out according to the manufactures’ manual and standard protocol [39]. Specific probes for *vanB* gene and the 23S rRNA gene of *E. faecium* were made by PCR DIG Probe Synthesis kit (Roche) and specific primers for each gene [40].

### Multilocus sequence typing (MLST) analysis

MLST was performed as previously described [41]. The house keeping genes *atpA*, *ddl*, *gdh*, *purK*, *gyd*, *pstS* and *adk* were sequenced and STs were determined according to MLST.net (http://efaecium.mlst.net/).

### Conjugation experiment

Filter mating was performed as described previously [42]. Briefly, an overnight culture of the bacteria was diluted 50-fold in 5 ml of fresh THB broth and pre-cultured until the end of the exponential phase. One hundred microliter of the donor culture and 100 μl of the recipient culture were mixed in 5 ml of THB broth. FA2–2 or BM4105RF and wild M1 or Y7 were used as the recipients and donors, respectively. The mating mixture of donor and recipient was collected on a 0.45 μm nitrocellulose membrane filter (Merck Millipore, Darmstadt, Germany), and incubated on THB agar plate at 37 °C for 5 h. The mating mixture was plated on selective THB agar containing rifampicin 25 mg/L, fusidic acid 25 mg/L and VAN 3 mg/L. After incubation at 37 °C for 48 h, the colonies that grew were isolated and purified at least twice. The frequency of conjugation was calculated as the number of transconjugants per donor cell.

### Isolation of the VAN-resistant revertants

Cultures of each strain grown in THB broth at 37 °C for 24 h were plated onto a THB agar plate with or without VAN at concentration of 16 mg/L. Colonies grown after 24 h of incubation at 37 °C were counted and the frequency of the rate of reversion was estimated from the colony-forming unit (CFU) ratio of resistant strains to total strains. The values were the average of three independent experiments with standard error. Colonies grown on THB agar plates with VAN (16 mg/L) were isolated on new THB agar plates containing VAN (16 mg/L). Single colonical isolations were performed at least twice for each strain. The MICs of the resulting revertants were determined by the agar dilution method as described above. The nucleotide sequences of the *vanB* gene cluster was determined as described above.

### qRT-PCR analysis

An overnight bacterial culture was diluted 100-fold in brain heart infusion (BHI) medium with or without VAN (1 mg/L) and incubated at 37 °C until exponential phase. After collection of the bacterial cells by centrifugation for 5 min at 12,000 rpm, total RNA was prepared using a Fast RNA Pro Red Kit (Q-Biogene, Inc) and Fast Prep disintegrator (40 s, speed: 6.0). The resulting RNA was further extracted with chloroform, precipitated with ethanol and resuspended in 0.05 ml diethylpyrocarbonate (DEPC)- treated water. Total RNA (30 μg) was incubated with recombinant DNase I (RNase-free) and RNase inhibitor (Takara Bio Inc., Shiga, Japan) at 37 °C for 30 min. After extraction with phenol-chloroform-isoamyl alcohol (25:24:1) and chloroform-isoamyl alcohol (24:1), the samples were precipitated with ethanol and resuspended with 20 μl of DEPC- treated water. The concentration of the RNA solution was determined by a fluorescence-based assay with Qubit 3.0 (Thermo Fisher Scientific Inc., MA). Reverse transcription was carried out with the PrimeScript RT Master Mix (Takara Bio Inc.). Real-time PCR was carried out with the Thunderbird SYBR qPCR Mix (Toyobo Co., Tokyo, Japan) using an ABI 7500 Fast RT-PCR instrument (ABI, CA). Real-time-PCR cycled at 1 min at 95 °C, followed by 40 cycles of 15 s at 95 °C, 15 s at 55 °C, 1 min at 72 °C. The real-time PCR primers were designed by Primer3Plus (http://www.bioinformatics.nl/cgi-bin/primer3plus/primer3plus.cgi) (Additional file 8: Table S3).

### Kinetics of cell growth

Overnight bacterial cultures were diluted 100-fold in fresh THB broth with or without VAN (1 mg/L). The culture was incubated at 37 °C and the turbidity was measured at an optical density of 620 nm at each time point using a Multiskan FC Microplate Photometer (Thermo Fisher Scientific Inc.).

### Plasmid construction and targeted mutagenesis of the *vanB* gene cluster

Targeted mutagenesis of the *vanS*_*B*_*, vanB, vanW* genes in the *vanB* gene cluster was performed as previously described [43]. Briefly, the DNA fragments to be inserted were constructed by PCR using the corresponding primers, as indicated in Additional file 8: Table S3, and inserted into the pCJK47 vector using the restriction enzyme *Eco*RI (Roche) and a DNA Ligation Kit (Takara Bio Inc.), as described previously. After transformation into *E. coli* EC1000 as previously described elsewhere, the recombinant-expressing *E. coli* strains were incubated in 5 ml of LB containing 300 mg/L erythromycin at 37 °C with shaking. Recombinant plasmid DNA was extracted using the QIAprep Spin Miniprep Kit (QIAGEN, Hilden, Germany). The constructed plasmids were sequenced to confirm that the desired sequence had been inserted. Electrotransformation into CK111/pCF10–101 was performed as previously described. Overnight cultures of the donor strain (CK111/pCF10–101, pCJK47-derivatives) and recipient strain (M1TC; *E. faecalis* FA2–2 transconjugants of M1 strain) were diluted 100-fold into fresh THB broth and incubated separately at 37 °C for 1 h. One hundred microliter aliquots of the donor and recipient cultures were mixed with 800 μl of fresh THB broth and incubated at 37 °C with shaking at 150 rpm for 12 h, and were then spread on a BHI plate (rifampicin 25 mg/L, fusidic acid 25 mg/L, erythromycin 10 mg/L and X-gal 100 mg/L). After incubation at 37 °C for 32–48 h, colonies that were blue in color were isolated and purified as these were expected to be integrant clones where the pCJK47-derivative plasmid had integrated into the chromosomal target locus of the recipient strain. These integrant clones were inoculated in THB and incubated at 37 °C for 12 h. After a 100-fold dilution, 100 μl of the culture broth was plated on an MM9YEG plate supplemented with 10 mM 4-Chloro-DL-phenylalanine (SIGMA-ALDRICH Co., MO) and 40 mg/L X-gal, followed by incubation at 37 °C for 12 h. White colonies were then isolated and purified as potential candidates carrying mutations within the target genes. The nucleotide sequences were checked to determine whether the desired nucleotide substitution had occurred in the target sequences.

## Additional files

Additional file 1: **Table S1.** Amino acid identities of *vanB* gene cluster of wild M1 and Y7 strains. ^*a*^The genetic information for VanB-type vancomycin resistant enterococci was obtained from the genome database in NCBI (http://www.ncbi.nlm.nih.gov/). MLG29 (accession no.; AY655721.2), UW7606x64/3 TC1 (accession no.; CP013009.1), V583 (accession no.; NC_004668), Aus0085 (accession no.; NC_021994.1), BM4661 (accession no.; FJ767776.1). ^*b*^Identities were calculated by ClustalW [45]. (XLSX 11 kb)

Additional file 2: **Figure S1.** Amino acid sequence alignment of VanS_B_ encoded by M1/Y7 and the other VanB-type VRE. The genetic information for VanB-type vancomycin resistant enterococci was obtained from the genome database in NCBI (http://www.ncbi.nlm.nih.gov/). Alignments of VanS_B_ amino acid sequence of wild M1/Y7 with typical VanB-type vancomycin resistant enterococci such as MLG229 (accession no.; AY655721.2), UW7606x64/3 TC1 (accession no.; CP013009.1), V583 (accession no.; NC_004668), Aus0085 (accession no.;NC_021994.1) and BM4661 (accession no.; FJ767776.1) were carried out using ClustalW. A box indicated the unique substitution to M1 /Y7 strains. (DOCX 116 kb)

Additional file 3: **Figure S2.** Amino acid sequence alignment of VanB encoded by M1/Y7 and the other VanB-type VRE. The genetic information for VanB-type vancomycin resistant enterococci was obtained from the genome database in NCBI (http://www.ncbi.nlm.nih.gov/). Alignments of VanB amino acid sequence of wild M1/Y7 with typical VanB-type vancomycin resistant enterococci such as MLG229 (accession no.; AY655721.2), UW7606x64/3 TC1 (accession no.; CP013009.1), V583 (accession no.; NC_004668), Aus0085 (accession no.;NC_021994.1) and BM4661 (accession no.; FJ767776.1) were carried out using ClustalW. A box indicated the unique substitution to M1 /Y7 strains. (DOCX 94 kb)

Additional file 4: **Figure S3.** Amino acid sequence alignment of VanW encoded by M1/Y7 and the other VanB-type VRE. The genetic information for VanB-type vancomycin resistant enterococci was obtained from the genome database in NCBI (http://www.ncbi.nlm.nih.gov/). Alignments of VanW amino acid sequence of wild M1/Y7 with typical VanB-type vancomycin resistant enterococci such as MLG229 (accession no.; AY655721.2), UW7606x64/3 TC1 (accession no.; CP013009.1), V583 (accession no.; NC_004668), Aus0085 (accession no.;NC_021994.1) and BM4661 (accession no.; FJ767776.1) were carried out using ClustalW. A box indicated the unique substitution to M1 /Y7 strains. (DOCX 85 kb)

Additional file 5: **Figure S4.** PFGE of S1 nuclease-treated DNA and hybridization with *vanB* gene probes. PFGE of S1 nuclease-treated DNAs isolated from M1, Y7, M1TC, Y7TC, FA2–2 and V583 was performed (Left) and separated DNAs were transferred to Nylon membrane by Southern blotting and hybridized to *vanB* gene probe (Right). Lanes: MM, Lambda Ladder PFG Marker (New England BioLabs, MA); 1, M1; 2, Y7; 3, M1TC; 4, Y7TC; 5, FA2–2; 6, V583. (DOCX 600 kb)

Additional file 6: **Figure S5.** PFGE of I-*Ceu*I-digested DNA and hybridization with 23srRNA gene and *vanB* gene probes. PFGE of I-*Ceu*I-digested DNAs isolated from M1, Y7, M1TC, Y7TC, FA2–2 and V583 was performed (Left) and separated DNAs were transferred to Nylon membrane by Southern blotting and hybridized to 23 s rRNA gene prove (Middle) and *vanB* gene probe (Right). Lanes: MM, Lambda Ladder PFG Marker (New England BioLabs, MA); 1, M1; 2, Y7; 3, M1TC; 4, Y7TC; 5, FA2–2; 6, V583. (DOCX 411 kb)

Additional file 7: **Table S2.** Mutations in intracytoplasmic domain of VanS_B_ and glycopeptides MICs of the VAN-resistant revertants. ^*a*^M1R1─M1R17; revertants from M1, Y7R1─7; revertants from Y7. ^*b*^The sequence was compared with the *vanB* gene cluster of the cognate parent strain, M1 or Y7. ^*c*^VAN: vancomycin, TEC: teicoplanin. N. D.; not detected. (XLSX 11 kb)

Additional file 8: **Table S3.** Oligonucleotides used in this study. ^*a*^Small letters indicate additional tag-nucleotides for plasmid construction to be digested by restriction. (XLSX 11 kb)

## Abbreviations

AMP: ampicillin
BAPS: Bayesian analysis of population structure
CFU: colony-forming unit
CHL: hloramphenicol
CIP: ciprofloxacin
CLSI: Clinical and Laboratory Standards Institutes
ERY: erythromycin
EUCAST: European Committee on Antimicrobial Susceptibility Testing for enterococci
GEN: gentamicin
KAN: kanamycin
MIC: minimum inhibitory concentration
MLST: multilocus sequence typing
PFGE: pulsed-field gel electrophoresis
SNV: single nucleotide variation
STR: streptomycin
TEC: teicoplanin
TET: tetracycline
VAN: vancomycin
VRE: vancomycin resistant enterococci
VVE: vancomycin variable *Enterococcus faecium*
