# Supplementary figures and images for: Dissemination and genetic analysis of the stealthy vanB gene clusters of Enterococcus faecium clinical isolates in Japan
Source: BMC Microbiol. 2018 Dec 13;18:213. doi: 10.1186/s12866-018-1342-1 (PMC6293572; doi:10.1186/s12866-018-1342-1)

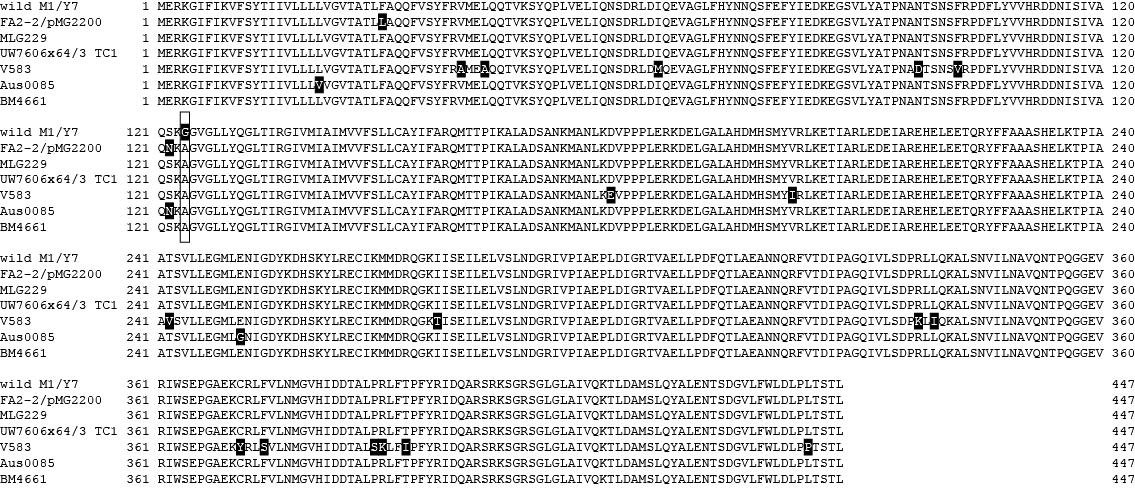

Supplement: Supplementary file 2 — Figure S1. Amino acid sequence alignment of VanSB encoded by M1/Y7 and the other VanB-type VRE. The genetic information for VanB-type vancomycin resistant enterococci was obtained from the genome database in NCBI (http://www.ncbi.nlm.nih.gov/). Alignments of VanSB amino acid sequence of wild M1/Y7 with typical VanB-type vancomycin resistant enterococci such as MLG229 (accession no.; AY655721.2), UW7606x64/3 TC1 (accession no.; CP013009.1), V583 (accession no.; NC_004668), Aus0085 (accession no.;NC_021994.1) and BM4661 (accession no.; FJ767776.1) were carried out using ClustalW. A box indicated the unique substitution to M1 /Y7 strains. (DOCX 116 kb) [file 12866_2018_1342_MOESM2_ESM.docx]

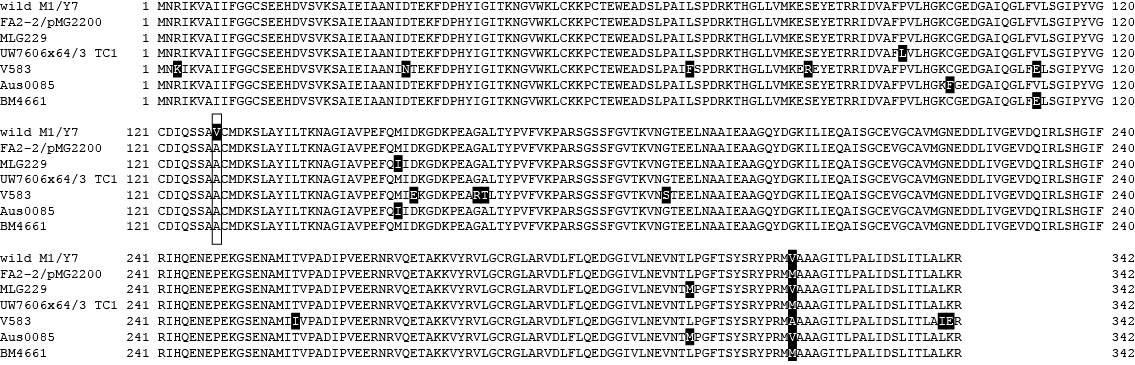

Supplement: Supplementary file 3 — Figure S2. Amino acid sequence alignment of VanB encoded by M1/Y7 and the other VanB-type VRE. The genetic information for VanB-type vancomycin resistant enterococci was obtained from the genome database in NCBI (http://www.ncbi.nlm.nih.gov/). Alignments of VanB amino acid sequence of wild M1/Y7 with typical VanB-type vancomycin resistant enterococci such as MLG229 (accession no.; AY655721.2), UW7606x64/3 TC1 (accession no.; CP013009.1), V583 (accession no.; NC_004668), Aus0085 (accession no.;NC_021994.1) and BM4661 (accession no.; FJ767776.1) were carried out using ClustalW. A box indicated the unique substitution to M1 /Y7 strains. (DOCX 94 kb) [file 12866_2018_1342_MOESM3_ESM.docx]

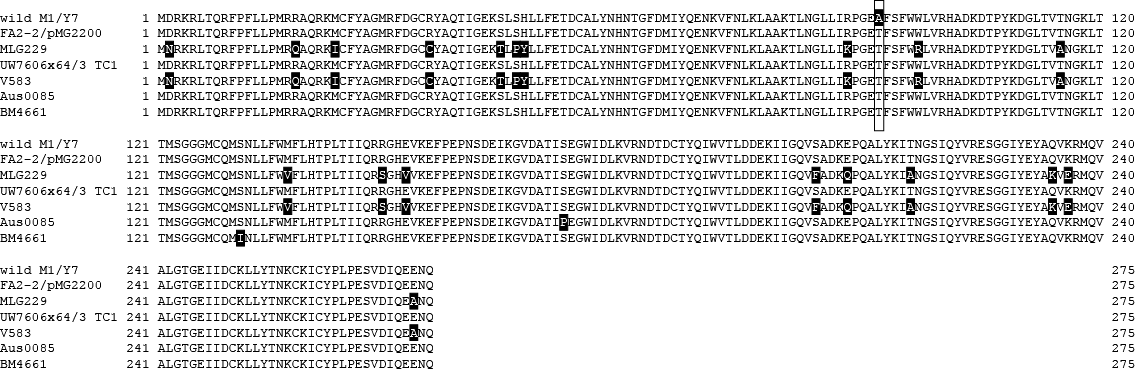

Supplement: Supplementary file 4 — Figure S3. Amino acid sequence alignment of VanW encoded by M1/Y7 and the other VanB-type VRE. The genetic information for VanB-type vancomycin resistant enterococci was obtained from the genome database in NCBI (http://www.ncbi.nlm.nih.gov/). Alignments of VanW amino acid sequence of wild M1/Y7 with typical VanB-type vancomycin resistant enterococci such as MLG229 (accession no.; AY655721.2), UW7606x64/3 TC1 (accession no.; CP013009.1), V583 (accession no.; NC_004668), Aus0085 (accession no.;NC_021994.1) and BM4661 (accession no.; FJ767776.1) were carried out using ClustalW. A box indicated the unique substitution to M1 /Y7 strains. (DOCX 85 kb) [file 12866_2018_1342_MOESM4_ESM.docx]

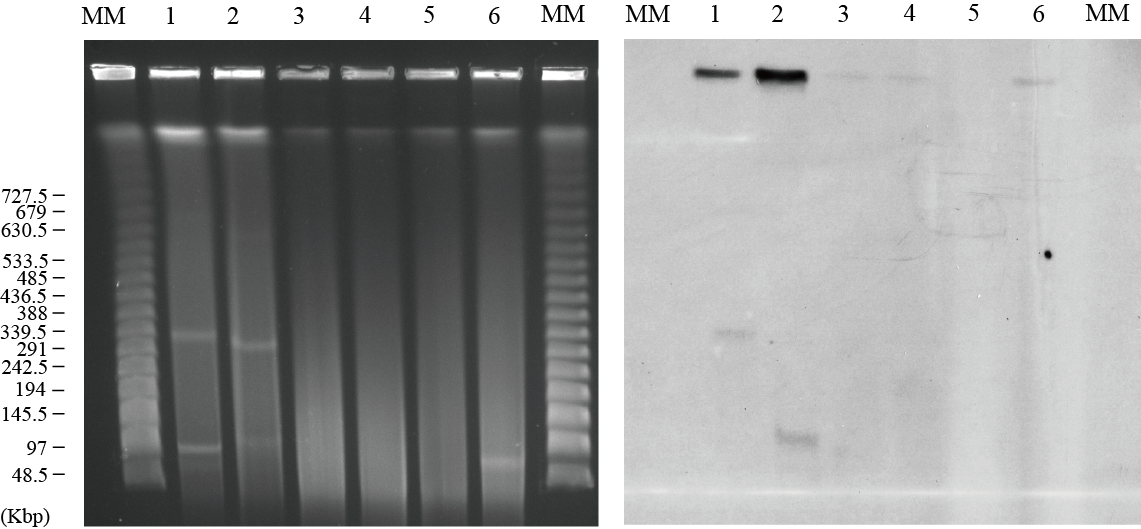

Supplement: Supplementary file 5 — Figure S4. PFGE of S1 nuclease-treated DNA and hybridization with vanB gene probes. PFGE of S1 nuclease-treated DNAs isolated from M1, Y7, M1TC, Y7TC, FA2–2 and V583 was performed (Left) and separated DNAs were transferred to Nylon membrane by Southern blotting and hybridized to vanB gene probe (Right). Lanes: MM, Lambda Ladder PFG Marker (New England BioLabs, MA); 1, M1; 2, Y7; 3, M1TC; 4, Y7TC; 5, FA2–2; 6, V583. (DOCX 600 kb) [file 12866_2018_1342_MOESM5_ESM.docx]

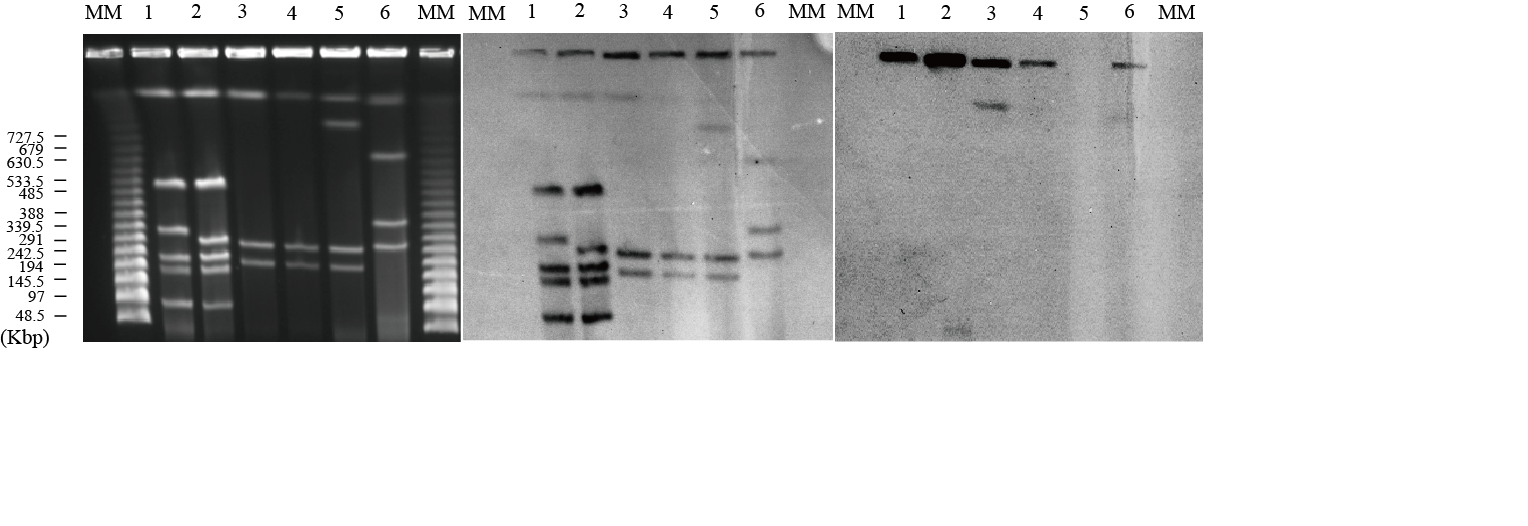

Supplement: Supplementary file 6 — Figure S5. PFGE of I-CeuI-digested DNA and hybridization with 23srRNA gene and vanB gene probes. PFGE of I-CeuI-digested DNAs isolated from M1, Y7, M1TC, Y7TC, FA2–2 and V583 was performed (Left) and separated DNAs were transferred to Nylon membrane by Southern blotting and hybridized to 23 s rRNA gene prove (Middle) and vanB gene probe (Right). Lanes: MM, Lambda Ladder PFG Marker (New England BioLabs, MA); 1, M1; 2, Y7; 3, M1TC; 4, Y7TC; 5, FA2–2; 6, V583. (DOCX 411 kb) [file 12866_2018_1342_MOESM6_ESM.docx]
